# Four-year follow-up of infliximab therapy in rheumatoid arthritis patients with long-standing refractory disease: attrition and long-term evolution of disease activity

**DOI:** 10.1186/ar2001

**Published:** 2006-07-17

**Authors:** Bert Vander Cruyssen, Stijn Van Looy, Bart Wyns, Rene Westhovens, Patrick Durez, Filip Van den Bosch, Herman Mielants, Luc De Clerck, Ann Peretz, Michel Malaise, Leon Verbruggen, Nathan Vastesaeger, Anja Geldhof, Luc Boullart, Filip De Keyser

**Affiliations:** 1 Department of Rheumatology, Ghent University Hospital, B-9000 Gent, Belgium; 2 Department of Electrical Energy, Systems and Automation, Ghent University, Gent, Belgium; 3 Department of Rheumatology, University Hospitals K.U. Leuven, Leuven, Belgium; 4 Department of Rheumatology, Cliniques Universitaires Saint-Luc, Brussels, Belgium; 5 Department of Rheumatology, University Hospital Antwerp, Antwerp, Belgium; 6 Department of Rheumatology, University Hospital Brugmann, Brussels, Belgium; 7 Department of Rheumatology, University Hospital Liège, Liège, Belgium; 8 Department of Rheumatology, AZ Vrije Universiteit Brussel, Brussels, Belgium; 9 Department of Medical Affairs, Schering-Plough, Brussels, Belgium; 10 Department of Medical Affairs, Centocor BV, Leiden, the Netherlands

## Abstract

Although there is strong evidence supporting the short-term efficacy and safety of anti-tumour necrosis factor-α agents, few studies have examined the long-term effects. We evaluated 511 patients with long-standing refractory rheumatoid arthritis treated with intravenous infusions of infliximab 3 mg/kg at weeks 0, 2, 6, and 14 and every 8 weeks thereafter for 4 years. Among the initial 511 patients included in the study, 479 could be evaluated; of these, 295 (61.6%) were still receiving infliximab treatment at year 4 of follow-up. The most common reasons for treatment discontinuation were lack of efficacy (65 patients, 13.6%), safety (81 patients, 16.9%), and elective change (38 patients, 7.9%). Analysis of disease activity scores (DAS28 [disease activity score based on the 28-joint count]) over time showed that, after the initial rapid improvement during the first 6 to 22 weeks of therapy, a further decrease in disease activity of 0.2 units in the DAS28 score per year was observed. DAS28 scores, measured at week 14 or 22, were found to predict subsequent discontinuation due to lack of efficacy. In conclusion, long-term maintenance therapy with infliximab 3 mg/kg is effective in producing further reductions in disease activity. Disease activity measured by the DAS28 at week 14 or 22 of infliximab therapy was the best predictor of long-term attrition.

## Introduction

After demonstration of effectiveness of anti-tumour necrosis factor (TNF)-α agents in patients with rheumatoid arthritis (RA) [1-3], their use has become common practice in treating patients with RA not responding to classical disease modifying anti-rheumatic drugs (DMARDs). Although there is strong evidence in support of the short-term efficacy and safety of these agents, data are still insufficient with regard to the long-term effects.

Long-term treatment continuation rates reflect safety, efficacy, and compliance to therapy and may vary between data from clinical trial extensions and treatment registries. Infliximab, primarily used in combination with methotrexate (MTX), is a highly effective therapy for the majority of patients with RA [4]. After an induction scheme with intravenous infliximab infusions given at weeks 0, 2, and 6, infliximab is typically administered at a dosage of 3 mg/kg every 8 weeks in combination with MTX. However, results of the ATTRACT (Anti-TNF Trial in Rheumatoid Arthritis with Concomitant Therapy) trial suggested that a higher dosage (10 mg/kg every 8 weeks) or a shorter perfusion interval may add benefit, which is reflected by the use of dosage increases in some studies [5,6].

In most countries, anti-TNF-α therapy is reserved for patients who are refractory to classical DMARD therapy. These patients may require TNF-α blockade for an extended time. We analysed data from patients who entered the Belgium expanded access program and received infliximab 3 mg/kg in combination with MTX. Patients in this program could receive infliximab therapy (provided by Schering-Plough, Brussels, Belgium) until the product became reimbursed. We aimed to (a) evaluate attrition of infliximab therapy in patients with long-standing refractory RA over a 4-year period, (b) document the reasons for discontinuation, (c) describe the long-term course of disease activity, and (d)evaluate early predictors of long-term continuation of the therapy.

## Materials and methods

### Study population

Five hundred eleven patients with RA entered the Belgium expanded access program between February 2000 and September 2001. These were the first Belgian patients to be treated with TNF blockade outside of the clinical trial setting after EMEA (European Medicines Evaluation Agency) approval of infliximab for the treatment of patients with RA and to receive infliximab from Schering-Plough for free as part of a Medical Need Program (the Belgian expanded access program) until the product became reimbursable.

Patients were observed at seven Belgian university centres. Clinical evaluations performed with each infliximab infusion included the 28 and 66/68 swollen and tender joint counts, erythrocyte sedimentation rate (ESR) (mm/hour), C-reactive protein (CRP) (mg/l), health assessment questionnaire ([HAQ] on a scale of 0–3) [7], physician's global assessment of disease activity using a visual analogue scale ([VAS] 0–100 mm), patient's global assessment of disease activity (VAS 0–100 mm), patient's assessment of pain (VAS 0–100 mm), patient's assessment of fatigue (VAS 0–100 mm), and all subscales of the short form (SF)-36 questionnaire (0–100 points) [8].

Along with the clinical evaluations performed on the day of each infusion, all physicians completed an evaluation of the 4-year experience. The evaluation provided an assessment of the actual therapy patients were receiving by year 4. If patients were withdrawn from infliximab therapy, the following information was collected: reasons for withdrawal (inefficacy, safety, death, or lost to follow-up); DAS28 [9,10]; physician's global VAS, CRP, and HAQ scores prior to infliximab withdrawal; and actual therapy at year 4.

All patients had long-standing, active, refractory RA. After an induction regimen of 3 mg/kg at weeks 0, 2, and 6, all patients received maintenance therapy every 8 weeks. At week 22, the treating rheumatologist had the option of increasing the dosage by 100 mg [11,12]. The standard infliximab dosage of 3 mg/kg every 8 weeks was reinstituted in a majority of patients beginning in June 2002, the time at which infliximab became a reimbursable medicine in Belgium. During the first 6 months, steroid and MTX dosages were kept stable; dosages could be adjusted from month 6 onward.

All patients gave written informed consent.

### Variables evaluated in the prediction of infliximab continuation

To predict long-term continuation of infliximab therapy early in the treatment course, we assessed the relationship between infliximab discontinuation and the following single variables at each of weeks 0, 6, 14, and 22: 28 and 66/68 swollen/tender joint counts, ESR, CRP, HAQ, physician's global assessment of disease activity, patient's global assessment of disease activity, patient's assessment of pain (VAS 0–100 mm), patient's assessment of fatigue, and all subscales of the SF-36 questionnaire. The patients' DAS28 scores and response (no, moderate, or good) and the American College of Rheumatology (ACR) response (no, 20, 50, or 70) were calculated after data collection so that the treating rheumatologist was unaware of the exact values of those composite scores [9,10,13].

### Statistical analysis

Statistical methods available in a classical statistical package (SPSS 12.0; SPSS Inc., Chicago, IL, USA) were employed in all analyses. The estimation of the slope of the course of disease activity from week 22 forward was estimated by means of linear mixed model analysis with an unstructured covariance matrix with random intercept and random slope [14]. Measurements from weeks 0, 6, 14, and 22 and the last clinical evaluation were included, as were available measures from other time points. When necessary, the continuous variables were normalised by taking the square root of the joint counts and the natural logarithm of CRP and ESR. Areas under the curve (AUCs) of receiver operating characteristic (ROC) curves were calculated. A higher AUC indicates that a single variable has better discriminative characteristics. The cutoff of the continuous variables was adapted to the same specificity level as the categorical variable so that sensitivities could be evaluated and compared between continuous and categorical variables [15]. The selection and comparison of variables by ROC-curve analysis was performed because this method gives a valid ranking of variables and does not depend on the number of patients available for that specific variable (in contrast to ranking methods based on *p *values) [16].

Survival analysis and predictors of continuation of infliximab therapy were analysed by means of Kaplan-Meier analysis and Cox regression. Cox regression analysis was performed with the default options of SPSS 12.0, and stepwise construction of models was performed by conditional forward and backward elimination using the strategy described by Hosmer and Lemeshow [17]. Survival data were calculated after censoring at 4 years.

## Results

### Continuation rates of infliximab therapy

**Figure 1:**
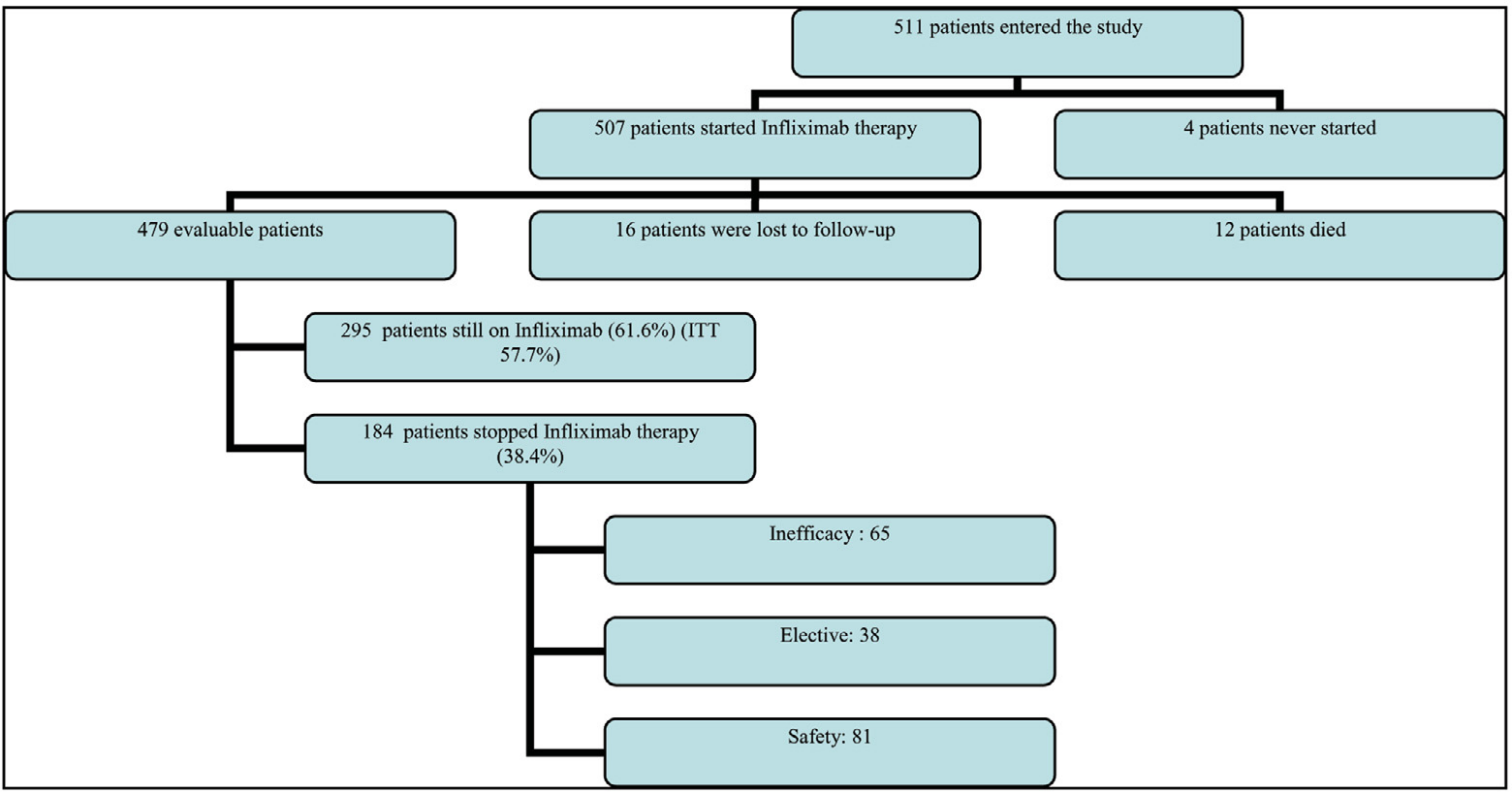
Flow chart of the patient population. ITT= intention to treat.

**Figure 2:**
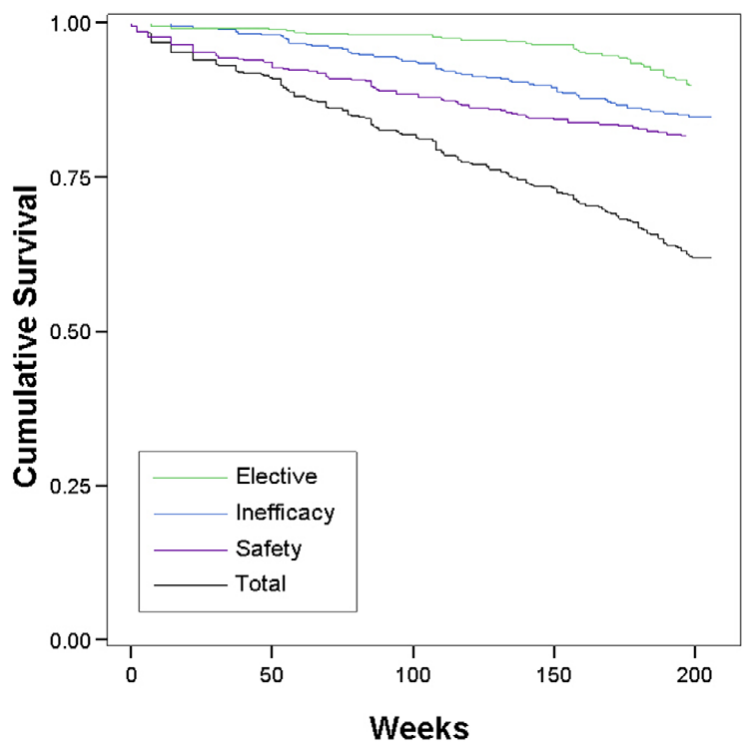
Kaplan-Meier plot and cumulative discontinuation due to the different stop reasons.

Of the initial 511 patients enrolled in the study, 507 effectively started infliximab therapy. All patients had long-standing, active, refractory RA, which is reflected by a mean baseline failure of 3.9 DMARDs and a mean disease duration of 10 years at baseline. After 4 years, 12 (2%) patients treated with infliximab had died (3 patients due to infections, 5 due to cardiovascular disease or lung embolism, and 4 due to other reasons; no patients died due to tuberculosis or anaphylactic reactions), and 16 (3%) patients were lost to follow-up. Of the 479 remaining patients, 295 (61.6%) patients were still receiving infliximab treatment after 4 years of therapy. One hundred eighty-four (38.4%) patients were withdrawn from treatment for the following reasons: 81 (16.9%) due to safety issues (including 28 infections, 18 immune-allergic reactions, and 9 malignancies), 65 (13.6%) due to inefficacy, and 38 (7.9%) for elective reasons (Figure 1). The main elective reason to stop infliximab treatment was the decision by the physician or the patient to switch to a subcutaneous TNF-α blocker. Those subcutaneous TNF-α blockers became available in February 2003 for etanercept and in May 2004 for adalimumab. Figure 2 provides the Kaplan-Meier plot of the attrition on infliximab therapy, illustrating the continuation rates after 1, 2, and 3 years; these data are also displayed in Table 1.

### Evaluation of the actual therapy at year 4 in the patients withdrawn from infliximab therapy

Data on the current DMARD or newly started biological therapy could be obtained in 142 of the 184 patients who discontinued infliximab therapy. Fifty percent of patients were switched to another biological therapy (Table 2).

### Evolution of the DAS28 score over time

**Figure 3:**
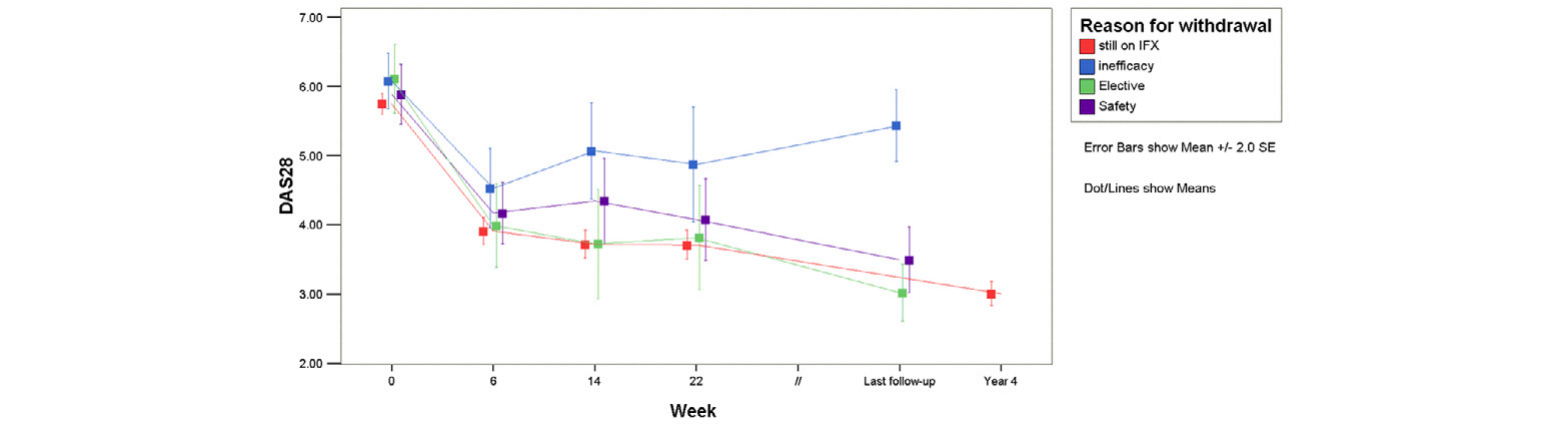
Evolution of the DAS28 (disease activity score based on the 28-joint count) scores and its components over time. DAS28 scores at the last clinical evaluation (evaluation at discontinuation of infliximab (IFX) treatment; median 119 weeks, interquartile range = 74 weeks) and the year 4 evaluation (median 205 weeks, interquartile range = 22 weeks). SE, standard error.

Patients continuing with infliximab therapy had a mean (standard deviation [SD]) DAS28 score of 3.0 (SD 1.3) at the year 4 clinical evaluation. In comparison, the mean DAS scores before the stop of therapy were 5.4 (SD 1.5) among patients who stopped due to inefficacy, 3.5 (SD 1.3) in patients who stopped due to safety reasons, and 3.0 (SD 1.0) in the patients withdrawn from infliximab for elective reasons. DAS28 scores at the relevant time points are depicted in Figure 3. This figure also suggests that, after an initial rapid decrease in disease activity between weeks 0 and 6, there appears to be a further decrease in disease activity through year 4. This decrease, calculated in the total population (all patients, including those who withdrew from therapy due to inefficacy), was estimated as a mean of 0.2 (standard error of the mean 0.03, *p *< 0.0001) DAS units per year. The same results were obtained in models that corrected for infliximab dosage and/or concomitant corticosteroid, MTX, or leflunomide use by adding them as (non-significant) covariates to the mixed models analysis.

Among the patients still receiving infliximab at year 4, 62% had a low level of disease activity, as defined by a DAS28 score of less than 3.2. Nearly half (49.5%) of these patients had minimal disease activity (DAS28 less than 2.85 or no swollen joints, no tender joints, and ESR less than 10 [18]). Of note, 17.4% of the patients had no swollen joints, no tender joints, and an ESR less than 10.

### Prediction of attrition of infliximab therapy

Ranking of all clinical evaluations conducted at weeks 0, 6, 14, and 22 showed that the DAS28 scores at weeks 14 and 22 are the most important measurements in predicting later withdrawal from infliximab therapy due to inefficacy, with ROC AUCs of 0.731 (standard error [SE] 0.06) and 0.706 (SE 0.06), respectively. None of the other parameters evaluated for prediction of treatment withdrawal had an AUC higher than 0.65, and they were therefore omitted from further evaluation. We combined in one Cox regression model the continuous DAS28 scores (at week 14 or week 22) with the response scores (ACR 20–50–70 response and no-moderate-good DAS response score). None of the models showed a significant additional value of those response scores to the continuous DAS28 alone. This indicates that the DAS28 at week 14 or 22 can predict long-term attrition better than DAS response or ACR response can.

The hazard ratio for withdrawal from infliximab therapy due to lack of efficacy was 1.9 (95% confidence interval [CI] 1.4–2.5) for high DAS scores at week 14 and was 1.7 (95% CI 1.3–2.1) for high DAS scores at week 22. These findings indicate that the likelihood of withdrawing from therapy increases by 70% to 90% when the DAS score increases by one unit.

**Figure 4:**
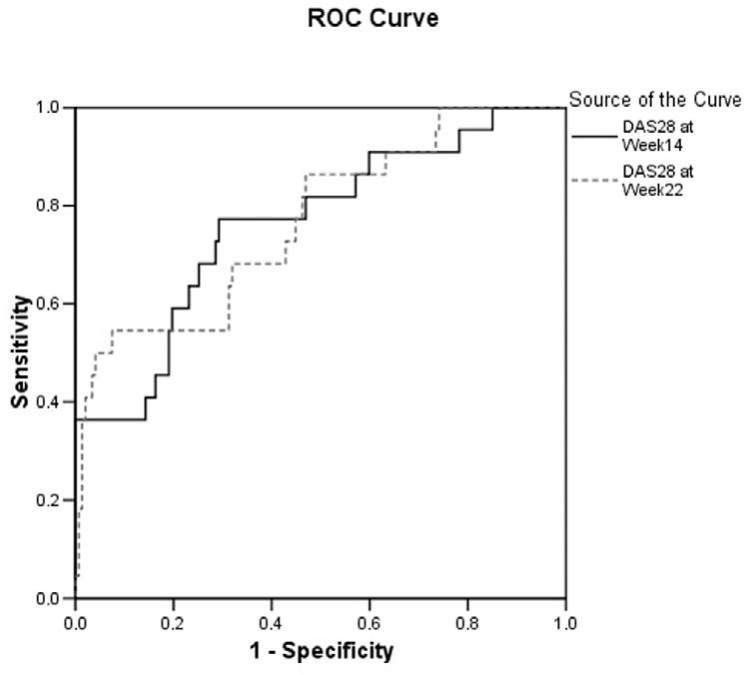
Receiver operating characteristic (ROC) analysis of DAS28 (disease activity score based on the 28-joint count) to predict withdrawal from treatment due to inefficacy.

To translate this hazard ratio in terms of sensitivity and specificity to predict withdrawal from therapy, we performed ROC curve analysis (Figure 4). At the 95% level of specificity, the week 14 and week 22 DAS28 scores show 35% and 37% sensitivities, respectively, of predicting withdrawal of infliximab therapy due to inefficacy (Table 3). Taking into account the 13.6% *a priori *chance to withdraw therapy due to inefficacy, a DAS28 of at least 6 at week 14 or 22 increases the probability of withdrawing from therapy to more than 50% (Table 3, Figure 4).

Transiently increasing the infliximab dose at week 22 in a subgroup of those patients with persistently high disease activity did not lead to a better attrition rate (data not shown).

## Discussion

In the present study, we prospectively evaluated the 4-year continuation rates and efficacy of infliximab in a large cohort of patients with long-standing refractory RA. Relatively few patients were lost to follow-up, which is important when estimating continuation rates and efficacy of therapy. After a 4-year study period, 61.6% of patients enrolled in the study were still receiving infliximab therapy. Over the same time period, 13.6% of patients discontinued infliximab therapy due to lack of efficacy, 16.9% due to safety issues, and 7.9% due to elective reasons. This is the first study that describes 4-year infliximab continuation rates in a large cohort of patients. Long-term continuation rates have also been reported for etanercept (25 mg) and adalimumab (40 mg) in open-label extensions of double-blind controlled trials [19,20]. In early RA and MTX-naïve patients who received etanercept, 63% of the 468 patients who entered the 3-year open-label extension were still receiving etanercept at the 5-year follow-up [19]. Similarly, 4 years after the initiation of the ARMADA (Anti-TNF Research Study Program of the Monoclonal Antibody D2E7 in Patients with RA) trial, 64% of the 271 enrolled patients were still receiving adalimumab therapy (mean duration of treatment = 3.4 years) [20].

Results of some smaller studies showed similar or lower infliximab continuation rates after 3 years (Voulgari *et al*. [21], *n *= 84, 59%), after 2 years (Geborek *et al*. [22], *n *= 135 75%; Wendling *et al*. [23], *n *= 41, 67%), and at 1 year (Flendrie *et al*. [24], *n *= 120, 58%; Zink *et al*. [25], *n *= 343, 65%; Chevillotte *et al*. [26], *n *= 60, 64%). Differences between results may be explained by differences in study populations and different availability of treatment alternatives.

Another manner of accessing long-term data is through national registry databases, which are primarily maintained to evaluate effectiveness and safety issues [27,28]. Evaluation of the efficacy of long-term therapy shows that, after 4 years of infliximab therapy, the majority of patients had a low level of disease activity and that approximately half of the patients met the criteria for minimal disease activity [18]. Moreover, we demonstrated that, after the initial rapid response to infliximab therapy between baseline and week 22, a further decrease in disease activity was observed over the remaining 3.5 years. This decrease in disease activity was observed in both the patients who continued with infliximab therapy and those who discontinued treatment later on due to safety or elective reasons. It is also important to mention that low levels of disease activity could be achieved and maintained with a standard infliximab dosage of 3 mg/kg and that dosage increases were transient in a majority of patients. The higher percentage of patients who need a dosage increase in some American studies might be explained by greater flexibility in the U.S. label or by a lower concomitant use of MTX in U.S. practice as compared with our study, in which 90% of the patients continue on infliximab + MTX combination therapy.

Also, transiently increasing the infliximab dose at week 22 in a subgroup of patients with a persistently high level of disease activity did not appear to affect the continuation rate.

We also assessed whether long-term response to infliximab therapy could be predicted early in the treatment protocol. Our findings suggest that persistently high levels of disease activity after an induction regimen of infliximab, as measured by the DAS28 score at week 14 or 22, was predictive of subsequent treatment discontinuation due to lack of efficacy. This observation corroborates the notion that a change in treatment strategy should be considered for patients with high levels of disease activity after 6 months of infliximab therapy. Switching to alternative therapies after 3 to 6 months if no therapeutic effect is observed is common in daily clinical practice and has been used in different studies to explore treatment options [29-31]. The optimal time point (that is, week 14 or 22) for determining whether a patient should continue therapy remains to be established and should take into account that predictive values may be highly influenced by the *a priori *chance to withdraw from treatment due to inefficacy, which was 13.6% in the present population. This *a priori *chance was modified to an *a posteriori *chance of 50% when the DAS28 at week 14 or 22 was higher than 6.

However, the data presented here clearly show that this decision is best made using the DAS28 score and not employing single measurements of swollen or tender joint count or response scores such as DAS28 response or ACR response score.

## Conclusion

The results of this study highlight that infliximab therapy is safe and effective for long-term (that is, 4 years) treatment of refractory RA. After an initial rapid response to the therapy, patients receiving infliximab continue to experience less disease activity over time. Our findings also indicate that the decision to continue infliximab therapy is best made using the DAS28 score.

## Abbreviations

ACR = American College of Rheumatology; AUC = area under the curve; CI = confidence interval; CRP = C-reactive protein; DAS28 = disease activity score based on the 28-joint count; DMARD = disease-modifying anti-rheumatic drug; ESR = erythrocyte sedimentation rate; HAQ = health assessment questionnaire; MTX = methotrexate; RA = rheumatoid arthritis; ROC = receiver operating characteristic; SD = standard deviation; SE = standard error; SF = short form; TNF = anti-tumour necrosis factor; VAS = visual analogue scale.

## Competing interests

The study was supported by a grant from Centocor BV and Schering-Plough. AG is an employee of Centocor BV, Leiden, the Netherlands. NV is an employee of Schering-Plough. PD and RW were consultants for Schering-Plough during the clinical study.

## Authors' contributions

BVC and SVL performed the statistical analysis, constructed the datasets, and drafted the manuscript. RW, PD, FVdB, HM, LDC, AP, MM, LV, and FDK recruited and observed the patients with arthritis. BVC, SVL, BW, NV, AG, LB, RW, PD, and FDK participated in the study design. RW and PD were the initial investigators of the Belgian infliximab expanded access program in which the patients were enrolled. All authors have read and approved the final manuscript.

## Figures and Tables

**Table 1:** Infliximab attrition rates at different time points

	Global continuation rates			Discontinuation rates								
				Inefficacy			Elective			Safety		
Year	Percentage	95% CI		Percentage	95% CI		Percentage	95% CI		Percentage	95% CI	
1	90.9	88.3	93.5	1.8	0.6	3.0	0.9	0.1	1.7	6.4	4.2	8.6
2	80.7	77.2	84.2	6.4	4.2	8.6	1.4	0.3	2.5	11.5	8.6	14.4
3	69.6	65.5	73.7	11.2	8.4	14.0	3.4	1.8	5.0	15.8	12.5	19.1
4	61.6	57.2	66.0	13.6	10.5	16.7	7.9	5.5	10.3	16.9	13.5	20.3

CI, confidence interval.

**Table 2:** Use of biologic agents after withdrawal of infliximab therapy

New biologic therapy	Reason for withdrawal			Total
	Lack of efficacy	Patient request	Safety issues	
None	24	3	44	71
Adalinumab	10	23	3	36
Etanercept	6	4	8	18
Rituximab	8	0	1	9
Abatacept	4	0	4	8
Total	52	30	60	142

**Table 3:** Sensitivity, PPV, and NPV of the DAS28 to predict infliximab discontinuation due to lack of efficacy

	DAS28 at week 14				DAS28 at week 22			
Specificity level	Cutoff	Sensitivity	PPV	NPV	Cutoff	Sensitivity	PPV	NPV
0.90	5.47	0.36	0.34	0.90	5.55	0.41	0.41	0.90
0.95	5.97	0.35	0.51	0.90	6.10	0.37	0.56	0.91
0.98	6.40	0.32	0.90	0.90	6.85	0.16	0.62	0.89

Sensitivities, PPVs, and NPVs are presented at different specificity levels of the DAS28 at week 14 or 22. DAS28, disease activity score based on the 28-joint count; NPV, negative predictive value; PPV, positive predictive value.
